# Nanoporous Carbon Derived from Green Material by an Ordered Activation Method and Its High Capacitance for Energy Storage

**DOI:** 10.3390/nano10061058

**Published:** 2020-05-30

**Authors:** Qingjie Lu, Shiqiang Zhou, Yumin Zhang, Mingpeng Chen, Bo Li, Haitang Wei, Dongming Zhang, Jin Zhang, Qingju Liu

**Affiliations:** 1 School of Materials and Energy, Yunnan Key Laboratory for Micro/nano Materials & Technology, International Joint Research Center for Optoelectronic and Energy Materials, Yunnan University, Kunming 650091, China; qjlu@mail.ynu.edu.cn (Q.L.); shiqiangzhou@mail.ynu.edu.cn (S.Z.); zhangyumin@ynu.edu.cn (Y.Z.); lb4869@mail.ynu.edu.cn (B.L.); htwei12345@126.com (H.W.); zhangdmyun@163.com (D.Z.); zhj@ynu.edu.cn (J.Z.); 2 Institute of Applied Physics and Materials Engineering, University of Macau, Macao, China; yb97809@um.edu.mo

**Keywords:** biomass carbon, two-step activation, supercapacitors, ultrahigh capacitance

## Abstract

Carbon materials have been widely used as electrode materials for supercapacitors, while the current carbon precursors are mainly derived from fossil fuels. Biomass-derived carbon materials have become new and effective materials for electrodes of supercapacitors due to their sustainability, low pollution potential, and abundant reserves. Herein, we present a new biomass carbon material derived from water hyacinth by a novel activation method (combination of KOH and HNO_3_ activation). According to the electrochemical measurements, the material presents an ultrahigh capacitance of 374 F g^−1^ (the current density is 1 A g^−1^). Furthermore, the material demonstrates excellent rate performance (105 F g^−1^ at a higher density of 20 A g^−1^) and ideal cycling stability (87.3% capacity retention after 5000 times charge–discharge at 2 A g^−1^). When used for a symmetrical supercapacitor device, the material also shows a relatively high capacity of 330 F g^−1^ at 1 A g^−1^ (a two-electrode system). All measurements suggest the material is an effective and noteworthy material for the electrodes of supercapacitors.

## 1. Introduction

The exploration of efficient and clean devices for energy storage has become an essential challenge for human beings. Supercapacitors have been considered to be promising candidates with advantages of long cycle life, high power density, wide working temperature range, high charge–discharge efficiency, and stability [1,2,3,4,5]. Electrode materials determine the electrochemical performance of supercapacitors, which combine the advantages of simple preparation processes, well-developed structures, vast resources, as well as being environmentally friendly [6,7,8,9,10]. Carbonaceous precursors have been mainly used as electrode materials because of their advantages of low cost, high specific surface area (SSA), reasonable structure, good electrical conductivity, and excellent chemical stability [11,12,13,14,15,16,17,18]. Porous carbon precursors have been explored for electrode materials of supercapacitors because their superb pore structures supply enough channels for ion transmission and provide sufficient active sites for electrochemical reactions, decreasing the transmission distance and further optimizing electrode efficiency and improving capacity. However, these precursors are usually derived from fossil fuels which are restricted by low-resource, as well as being environmentally unfriendly and unsustainable [19,20,21,22]. 

Green biomass-derived carbon materials have raised hopes because of their abundant reserves, low pollution, sustainability, and applicability. To date, many kinds of biomass sources including animals and plants have been explored as precursors for carbon-based electrode materials [23,24,25,26,27]. In general, biomass carbonaceous materials display manifold microcosmic morphologies, such as tubes, fibers, spheres, and sheets [28,29,30,31,32,33,34,35]. These biomass carbon materials applied for supercapacitors exhibit many advantages of well-distributed pore size, ultrahigh SSA, stable surface chemical properties, and high electric conductivity, which make them promising materials for electrodes of supercapacitors [36,37]. Although much progress has been achieved for biomass carbon used as electrode materials, the application and developments are limited by several challenges and shortcomings. For example, the surface properties of biomass-derived carbon materials, such as pore structure and SSA are hard to regulate. Generally, ultrahigh SSA is very conducive for the electrochemical behavior of the biomass-derived carbon precursors, but vast micropores are generated during the chemical activating processes for improving the SSA, which are not effective for the transmission of ions [38,39,40,41]. Moreover, the introduction of heteroatoms benefits electrochemical reactions by modifying internal structural properties and surface characteristics of the carbon precursors, such as increasing the interlayer spacing and active sites. However, the electric conductivity of the precursors is decreased, which is an adverse effect for electrochemical behavior. All these challenges and shortcomings need to be solved for their further applications in supercapacitor fields [42,43].

Herein, a new biomass carbon derived from water hyacinth by an ordered activation method (combination of KOH activation and HNO_3_ activation) is reported, which shows a superb electrochemical capacitance performance for supercapacitors. Generally, the HNO_3_ activation process is first undertaken after a direct carbonization treatment (the sample prepared by direct carbonization denoted as water hyacinth-derived carbon: WHC), some pores are generated as well as the SSA value is increased, and the obtained material by this activation is denoted as activated water hyacinth-derived carbon (AWHC). Subsequently, the KOH activation process was undertaken, in which vast micropores and mesopores could be generated. The obtained final sample which is denoted as double activated water hyacinth-derived carbon (AAWHC) possesses an ultrahigh SSA of 1622.6 m^2^ g^−1^ and vast mesopores and micropores. AAWHC shows an ultrahigh specific capacitance of 374 F g^−1^ (when used for electrode materials of supercapacitors, in a three-electrode system) at 1 A g^−1^ and a long cycle life of 87.3% retention (5000 times charge-discharge) at a current density of 2 A g^−1^.

## 2. Preparations and Measurements

### 2.1. Preparation of Activated Water Hyacinth-Derived Carbon (AAWHC) 

An amount of 100 g water hyacinth obtained from a pond was rinsed more than once with absolute ethyl alcohol and deionized water, and dried in an oven at 70 °C for 24 h to obtain the dried water hyacinth leaves. Subsequently, the leaves were carbonized directly at 800 °C for 12 h to acquire the WHC. Then the mixtures of WHC and 98% HNO_3_ (Reagent. Tianjin Fengchuan Co. Ltd., Tianjin, China) (1 g:20 mL) were stirred at normal temperature for a whole day. Next the achieved mixtures were washed several times with deionized water, then heat treated in a tubular furnace at 800 °C for 2 h. After cooling to normal temperature, samples were treated by washing and drying processes to obtain AWHC, the above corresponds to step one of the activation process. The following is step two of the activation process. Mixtures of AWHC and KOH (Reagent. Tianjin Fengchuan Co. Ltd., Tianjin, China) (a mass ratio of 1:3) were fully ground and then treated in a tubular furnace at 800 °C for 2 h. After cooling to room temperature, 1 M HCl (Reagent. Tianjin Fengchuan Co. Ltd., Tianjin, China) aqueous solution was mixed with the sample with stirring for 4 h to wash away the redundant KOH and then rinsed repeatedly with deionized water until the pH changed to 7.0, before being dried to achieve the final sample AAWHC (according to the later results of XPS characterization, there was no Cl element measured, which confirms the effect of HCl is only washing, with no activation effect). 

### 2.2. Characterization

Differential scanning Calorimetry (DSC) and Thermogravimetry (TG) tests were conducted by a thermal analyzer (STA ZCT-B, Jingyi Co. Ltd., Beijing, China) at a temperature of 0–1000 °C (heating rate: 10 °C min^−1^). The element compounds were measured using X-ray photoelectron spectroscopy (XPS, K-Alpha spectrometer, Tgermo Fisher Scientific Co. Ltd., Lexington, MA, USA) with Al Ka excitation (1486.6 eV). Raman patterns were tested using a laser Raman microscope (Renishaw Co. Ltd., Birmingham, UK) (wave-length of argon ion laser: 633 nm). The pore properties were measured with a Barrett–Joyner–Halenda (BJH) method and SSA was detected with a Brunauer–Emmett–Teller (BET) method. Scanning electron microscope (SEM, S-3400N, HitachiCo. Ltd., Tokyo, Japan) and transmission electron microscope (TEM, JEM-2100, JEOL, Tokyo, Japan) were conducted to analyze the microcosmic morphology and structures. 

### 2.3. Electrochemical Measurements

Preparation of working electrodes: Samples WHC, AWHC, and AAWHC were separately blended with polytetrafluoroethylene (PTFE) and carbon black in mortar (mass ratio: 8:1:1), then pure ethanol was mixed, and the mixture ground adequately to achieve a slurry before smearing immediately on a pure nickel foam. The nickel foam was washed by HCl, deionized water, and absolute ethyl alcohol several times with ultrasound to ensure purity. The foams were dried in an oven at 120 °C for a whole day and treated with a tableting process to obtain the working electrodes.

Measurement conditions: For the measurements of capacitance behaviors of WHC, AWHC, and AAWHC, the electrochemical workstation CHI760 was utilized for galvanostatic charge–discharge (GCD), cyclic voltammetry (CV), and electrochemical impedance spectroscopy (EIS) measurements. Specifically, an Ag/AgCl electrode was prepared as reference electrode and a platinum film electrode as counter electrode, the electrolyte was 6 M KOH aqueous solution. In a three-electrode system, the GCD measurements were conducted with a potential range of −0.8 to 0.2 V at current densities of 1, 2, 5, 10, 20 A g^−1^. CV tests were conducted with a potential range of −0.8 to 0.2 V vs. Ag/AgCl electrode at different scan rates of 5, 10, 20, 50, 100 mV s^−1^. The EIS tests were conducted with a frequency range of 0.01Hz to 100 KH_Z_ (amplitude: 5 mV). Moreover, a simple supercapacitor device was constructed by utilizing two identical AAWHC electrodes and measured with a two-electrode system in 6M KOH solution. Besides, cycle measurements, the values of the charge and discharge time were calculated (5000 cycles at 20 A g^−1^). In a three-electrode system, capacitance C is calculated by GCD data based on the formula:

(1) C=(i·Δt)/(m·ΔV)

where S (V s^−1^) is the scan rate, i (A) is the response current value, ΔV (V) is the potential window in the measurements, m (g) is the mass value of the electrode materials smeared on pure nickel foam, Δt (s) is the discharging time.

For these assembled devices, capacity value Cs, energy density E, and power density P are calculated from GCD data detected in the two-electrode system based on these formulas:

(2) Cs=2(i·Δt)/(m·ΔV)

(3) E=(Cs·ΔV)/(8×3.6)

(4) P=E/Δt

where Cs (F g^−1^) is the capacity value, Δt (s) is the discharging time, m (g) is the mass value of the electrode materials on pure nickel foam, i (A) is the response current, ΔV (V) is the potential range, P (W Kg^−1^ ) is the specific power density, and E (Wh Kg^−1^ ) is the specific energy density.

## 3. Results and Discussion

**Figure 1:**
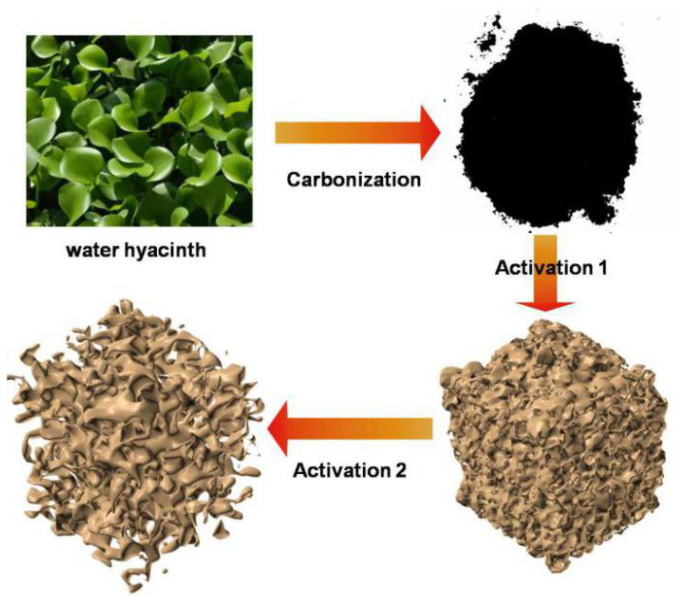
The scheme of the preparation process.

The preparation processes are shown in Figure 1. Briefly, water hyacinth was carbonized and the two-step activation followed (HNO_3_ activation precedes KOH activation). This scheme was also used for presenting the variations in structure (gradually looser) and porosity (increase of pores). The HNO_3_ activation process promotes some complex reactions which can increase the polarity, pores and SSA, indicating sufficient ion transmission channels and electrochemical active sites [44,45]. The KOH activation process optimizes the pore structure and increases the SSA of the biomass-derived carbon precursors by removing the non-carbon atoms, etching the surface, and generating vast pores [46,47,48]. The followed electrochemical measurements confirmed the right choice of the activation order (Figure S1). According to TG and DSC curves (Figure S2a,b), heat release and weight loss took place at temperatures of 0–100 °C and this corresponded to dehydration. Between 100 °C and 500 °C, AAWHC displays a strong endothermic peak with no weight loss, indicating phase transition. After 500 °C, AAWHC displays a rapid exotherm with rapid weight loss, indicating reactions between oxygen gas and AAWHC.

**Figure 2:**
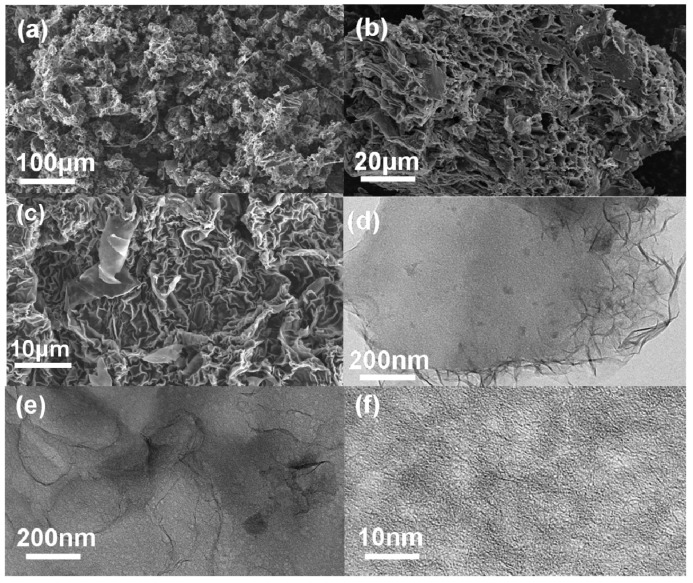
(**a**–**c**) SEM images of double activated water hyacinth-derived carbon (AAWHC). (**d**–**f**) TEM images of AAWHC.

SEM and TEM were used to explore the morphology and microstructure of samples. As shown in Figure 2a–c, SEM images of AAWHC display a loose bulk assembled structure with abundant channels and some wrinkles. Compared with WHC and AWHC, a looser and more porous structure is revealed. According to Figure S3a–c, the bulk structures of the samples mainly became increasingly looser after the ordered HNO_3_ activation and KOH activation. As shown in Figure S3d,e, there are no obvious changes apparent, which is due to the mechanism of HNO_3_ activation; no vast pores are generated to significantly change the structure morphology. As shown in Figure S3f, the generation of pores and wrinkles are confirmed in the same TEM scale. Clearly, From Figure 2d,e, there are some wrinkles. From Figure 2e,f, vast pores with different pore sizes can be observed. The above microstructure and morphology characteristics such as abundant channels, wrinkles and vast pores are conducive to rapid electrolyte ion transmission, providing sufficient active sites and improving the SSA for electrochemical redox reactions, thereby further enhancing the electrochemical performance of the samples [49,50,51].

**Figure 3:**
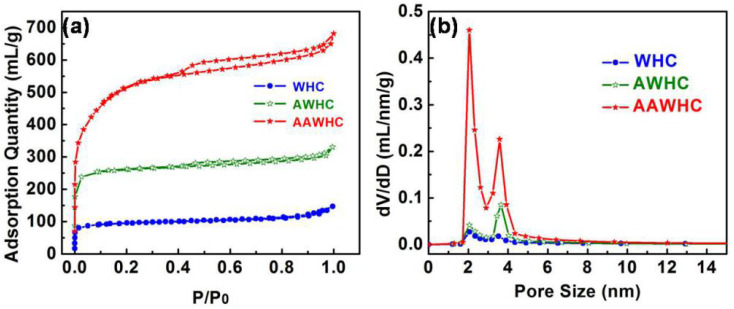
(**a**) N_2_ adsorption/desorption isotherms of the samples. (**b**) The pore size distribution of the samples.

The SSA and pore properties of all samples were measured by the N_2_ adsorption/desorption system. As shown in Figure 3a, steeper adsorptions at low pressure indicate that all the samples possess abundant micropores, which improve the SSA and provide enough active sites for electrochemical reactions. At high relative pressure areas, the distinct hysteretic curves demonstrate the existence of mesopores and it is very apparent that the final sample AAWHC possesses the most obvious hysteretic curve. As shown in Figure 3b, AAWHC has a more reasonable pore distribution, the high content of mesopores will be conducive to its capacity performance. The structure properties and SSA of all the samples are summarized in Table 1. Intuitively, AAWHC exhibits an ultrahigh SSA of 1623 m^2^ g^−1^ while the value of AWHC is 740 m^2^ g^−1^ and WHC is 350 m^2^ g^−1^. AAWHC possesses a higher mesopore content (0.3962 mL g^−1^) than that of AWHC (0.1127 mL g^−1^) and WHC (0.0856 mL g^−1^), which means the pore size distribution of AAWHC is more reasonable. When used as electrode materials, sufficient micropores and mesopores as well as high SSA can provide sufficient electrolyte ion transmission channels and active sites for electrochemical reactions, further accelerating electrode efficiency [52,53,54].

**Figure 4:**
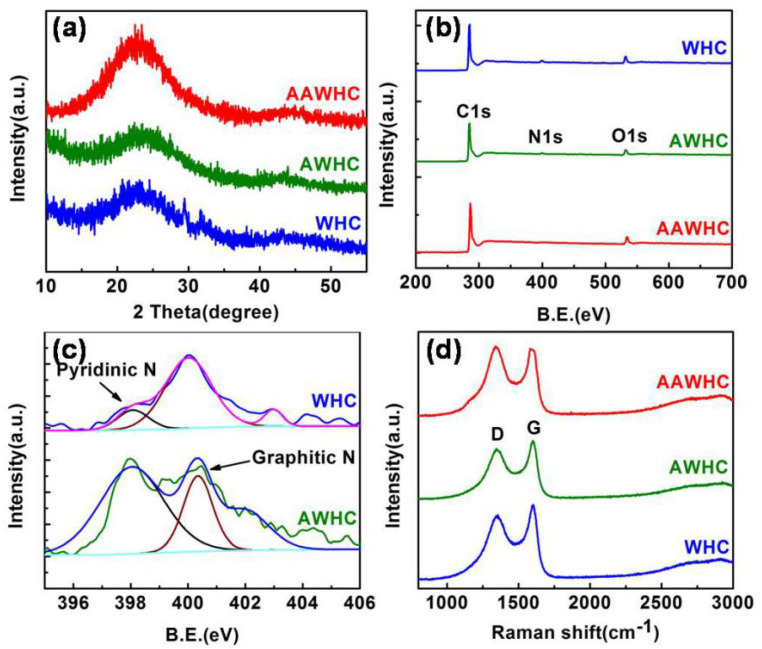
(**a**) XRD curves of the samples. (**b**) XPS curves of the samples. (**c**) N1s spectra of the samples at high resolution. (**d**) Raman curves of the samples.

The crystalline structures of the samples were confirmed by analyzing the XRD patterns. According to Figure 4a, the three patterns demonstrate a disordered structure (a basic characteristic of amorphous carbon) of the samples. Specifically, a wide diffraction peak at ~22° demonstrates the graphitic part of the biomass-derived carbon precursors. It can be seen that all diffraction peaks become wider and weaker on increasing the 2 theta value because of the generation of vast micropores (three samples are the same), and also the degree of disorder of the carbon precursors increases [55,56]. XPS patterns were examined to detect the elements in the samples. According to Figure 4b, the N1s peaks showed a nitrogen content of 3.14 at% in the WHC, and a value of 4.08 at% in the AWHC, and nearly zero in the AAWHC. Two main peaks can be observed at 398.3 eV (pyridinic nitrogen) and 401.0 eV (graphitic nitrogen) in the high-resolution patterns of the N1s spectra (Figure 4c). Obviously, the XPS curves of WHC, AWHC, AAWHC demonstrate that some N atoms were introduced into AWHC by the first activation (nitric acid activation), but almost the entire amount of N atoms was removed by the KOH activation process. Briefly, compared with WHC, AWHC possesses a higher nitrogen content, the doped N atoms can improve the active sites, pore size, and interlayer spacing to accelerate electrolyte ion transmission [57,58]. To further confirm the structural features of all samples, Raman spectra was conducted. As shown in Figure 4d, two main peaks are obvious: the D (defect) peak at ~1353 cm^−1^ and G (graphite) peak at ~1588 cm^−1^ [59]. The intensity ratio I_D_/I_G_ of AAWHC (1.01) is higher than that of WHC (0.88) and AWHC (0.93), indicating that AAWHC samples contain more edges and defect sites, which are beneficial for electrochemical reactions [60,61]. 

**Figure 5:**
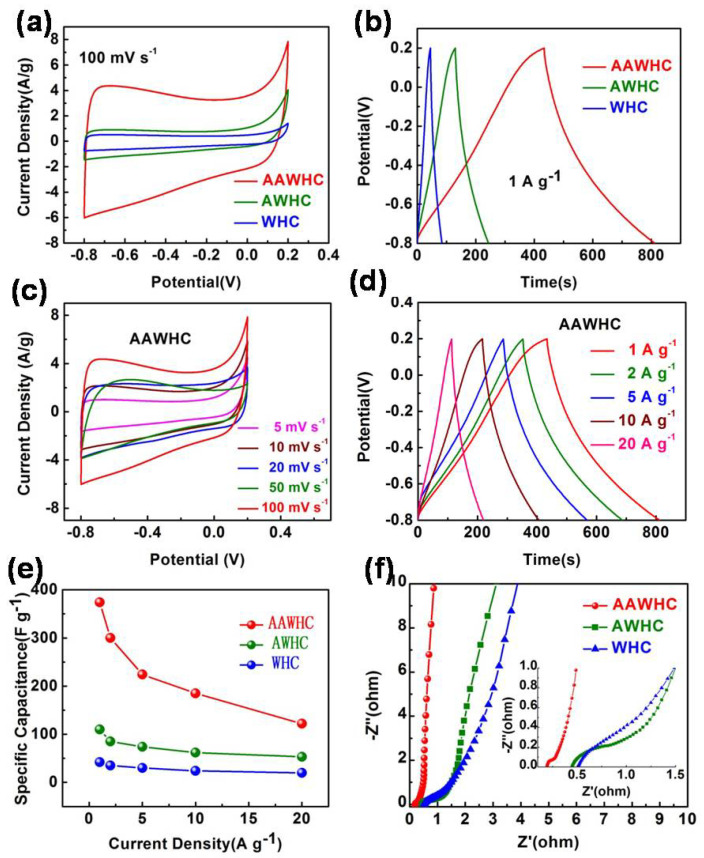
Electrochemical tests in a three-electrode system. (**a**) CV curves of the samples at a scan rate of 100 mV s^−1^. (**b**) GCD curves of the samples at a current density of 1 A g^−1^. (**c**,**d**) CV and GCD curves of AAWHC at different scan rates and current densities, respectively. (**e**) Specific capacitance of the samples at different current densities. (**f**) Nyquist plots of the samples.

To measure the electrochemical performance of all samples, EIS, GCD, and CV measurements were conducted and integrated into the three-electrode system. According to the CV curves of all samples at 100 mV s^−1^ (Figure 5a), it is obvious that AAWHC has a much larger rectangular-like curve than that of AWHC and WHC, which indicates AAWHC would exhibit a much higher specific capacity. Based on the GCD curves of samples (Figure 5b), all the curves display symmetrically triangular features and AAWHC has a much longer discharge time than AWHC and WHC, demonstrating the superb capacity ability of AAWHC. Calculating from the GCD curves, AAWHC possesses an ultrahigh specific capacitance (374 F g^−1^) and the values for AWHC and WHC are 114 F g^−1^ and 47 F g^−1^, respectively. However, the pure nickel foam possesses a basic capacitance, so the pure nickel foam underwent the additional GCD test—the measured capacitance value suggests this has no influence on the accuracy of the calculation results (Figure S4). According to Figure 5c, CV curves of AAWHC exhibit stable rectangular-like patterns at 5, 10, 20, 50, 100 mV s^−1^, demonstrating the ideal retention performance of AAWHC. Figure 5d shows the GCD patterns of AAWHC at 1, 2, 5, 10, 20 A g^−1^, maintaining good symmetrically triangular features and indicating a good rate performance of AAWHC [62]. Specifically, AAWHC still possesses an ideal capacity of 105 F g^−1^ at the higher current densities of 20 A g^−1^. The capacity of all samples at diverse densities are displayed in Figure 5e. It is obvious that AAWHC displays an outstanding rate capability, indicating that AAWHC is a promising candidate for electrode materials of supercapacitors. The electrochemical performance comparison with the previous reports is summarized in Table S1. The following EIS measurements were conducted to detect the charge and discharge transmission resistance of all samples. As shown in Figure 5f, AAWHC exhibits a more vertical curve than AWHC and WHC at a relative lower frequency range, suggesting a better capacitive behavior. Moreover, the semicircle of AAWHC is smaller than AWHC and WHC at a higher frequency range, indicating lower charge and discharge transmission resistance and leading to faster ion transportation of electrochemical reactions [63]. Calculating with the EIS data, AAWHC possesses lower electrolyte resistance (Rs, 0.69 Ω) and charge transfer resistance (Rct, 0.19 Ω) than the values for AWHC (Rs, 0.76 Ω; Rct, 0.44 Ω) and WHC (Rs, 1.06 Ω; Rct, 0.53 Ω).

**Figure 6:**
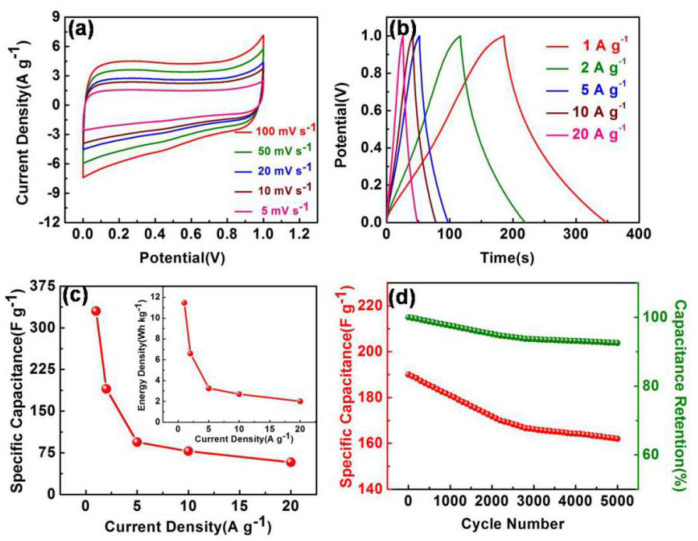
Electrochemical performances in a two-electrode system, 6 M KOH aqueous solution used for the electrolyte. (**a**) CV curves and (**b**) GCD curves of the device at different scan rates and current densities, respectively. (**c**) Energy densities and specific capacitance of AAWHC at different current densities. (**d**) Cycling stability at 2 A g^−1^ (5000 cycles).

Two identical AAWHC electrodes were constructed in a simple symmetrical supercapacitor device (electrolyte: 6.0 M KOH solution). According to the CV curves of the AAWHC-based device (Figure 6a), which display well-maintained rectangular patterns, it presented a superb rate performance. GCD curves measured at 1, 2, 5, 10, 20 A g^−1^ are displayed in Figure 6b, showing the typical triangular nature, indicating that the symmetrical device has promising electrical double layers at the interface of electrode/electrolyte and ideal rate performance. Calculating from the curves, the devices show a high capacity of 330 F g^−1^. A Ragone plot summarizes the capacitance of the device at 1, 2, 5, 10, 20 A g^−1^ (Figure 6c), and the Figure 6c inset reveals that the device displays a relative high specific energy density of 11.46 Wh kg^−1^ at 1 A g^−1^ and according to Figure S5, a superb energy density was confirmed. Besides, the AAWHC electrodes display a long cycle life at 2 A g^−1^ (Figure 6d), the capacity value of AAWHC remains at 164 F g^−1^ after 5000 times charge and discharge (12.7% loss). All the results demonstrate that AAWHC offers excellent energy density and capacity behaviors, probably due to the excellent surface chemical properties and structural characteristics of AAWHC. 

## 4. Conclusions

To summarize, a new mesopore-rich nanoporous biomass carbon AAWHC derived from water hyacinth was fabricated by an innovative preparation method which combined HNO_3_ activation and KOH activation. The as-prepared materials display a loose bulk structure with abundant channels and some wrinkles. Acting as electrode material for a supercapacitor, AAWHC shows a superb specific capacity of 374 F g^−1^ at a 1 A g^−1^ and a promising rate performance. Moreover, AAWHC shows a high specific energy density of 11.46 Wh kg^−1^ and an excellent cycle life (87.3% capacity retention after 5000 times charge and discharge) in a symmetric supercapacitor device. It is certain that with the superiority displayed in this paper, such a mesopore-rich carbon material which possesses advantages of low-cost and sustainability will have promising potential for energy storage applications.

## Figures and Tables

**Table 1:** Pore structure properties of samples (SSA: Specific Surface Area).

Samples	SSA/m^2^ g^−1^	Pore Volume/mL g^−1^	Mesopore Volume/mL g^−1^
WHC	352	0.2288	0.0856
AWHC	741	0.5116	0.1127
AAWHC	1623	1.0547	0.3962

## Supplementary Materials

The following are available online at https://www.mdpi.com/2079-4991/10/6/1058/s1, Figure S1: GCD curves of the samples activated in a different order, Figure S2: (**a**) DSC curve of AAWHC. (**b**) TG curve of AAWHC, Figure S3. (**a**) SEM image of WHC. (**b**) SEM image of AWHC, Figure S4. GCD curve of the pure nickel foam, Table S1. Comparison with the latest reports of biomass carbon.
