# Supplementary material for: Nanoporous Carbon Derived from Green Material by an Ordered Activation Method and Its High Capacitance for Energy Storage
Source: Nanomaterials (Basel). 2020 May 30;10(6):1058. doi: 10.3390/nano10061058 (PMC7352300; doi:10.3390/nano10061058)
Supplement: Supplementary file 1 [file nanomaterials-10-01058-s001.pdf]

nanomaterials

Supporting Information

Nanoporous Carbon Derived from Green Material by an Ordered Activation Method and Its High Capacitance for Energy Storage

Qingjie Lu 1, Shiqiang Zhou [1], Yumin Zhang [1], Mingpeng Chen [2], Bo Li [1], Haitang Wei [1], Dongming Zhang [1], Jin Zhang [1] and Qingju Liu [1,*]

1 School of Materials and Energy, Yunnan Key Laboratory for Micro/nano Materials & Technology, International Joint Research Center for Optoelectronic and Energy Materials, Yunnan University, Kunming 650091, China; qjlu@mail.ynu.edu.cn (Q.L.); qjlu@mail.ynu.edu.cn (S.Z.); zhangyumin@ynu.edu.cn (Y.Z.); lb4869@mail.ynu.edu.cn (B.L.); htwei12345@126.com (H.W.); zhangdmyun@163.com (D.Z.); zhj@ynu.edu.cn (J.Z.)

2 Institute of Applied Physics and Materials Engineering, University of Macau, Macao SAR, China; yb97809@um.edu.mo

* Correspondence: qjliu@ynu.edu.cn

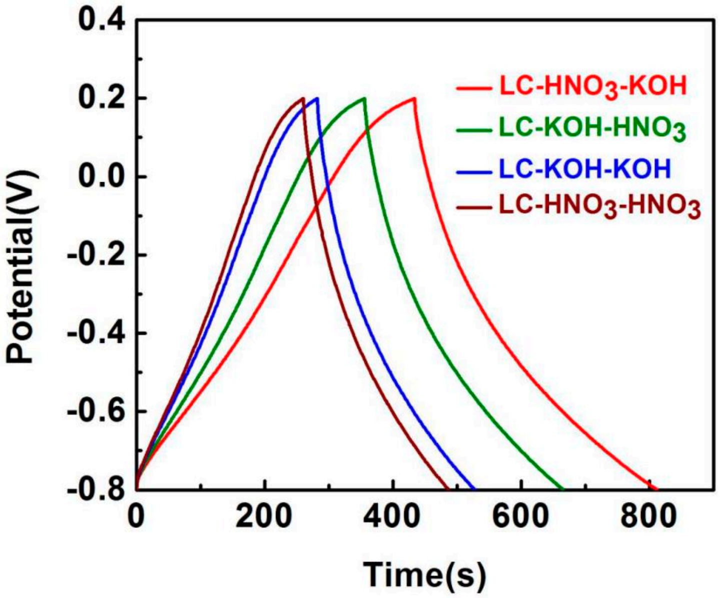

Figure S1. GCD curves of the samples activated in a different order at a current density of 1 A g^{-1}.

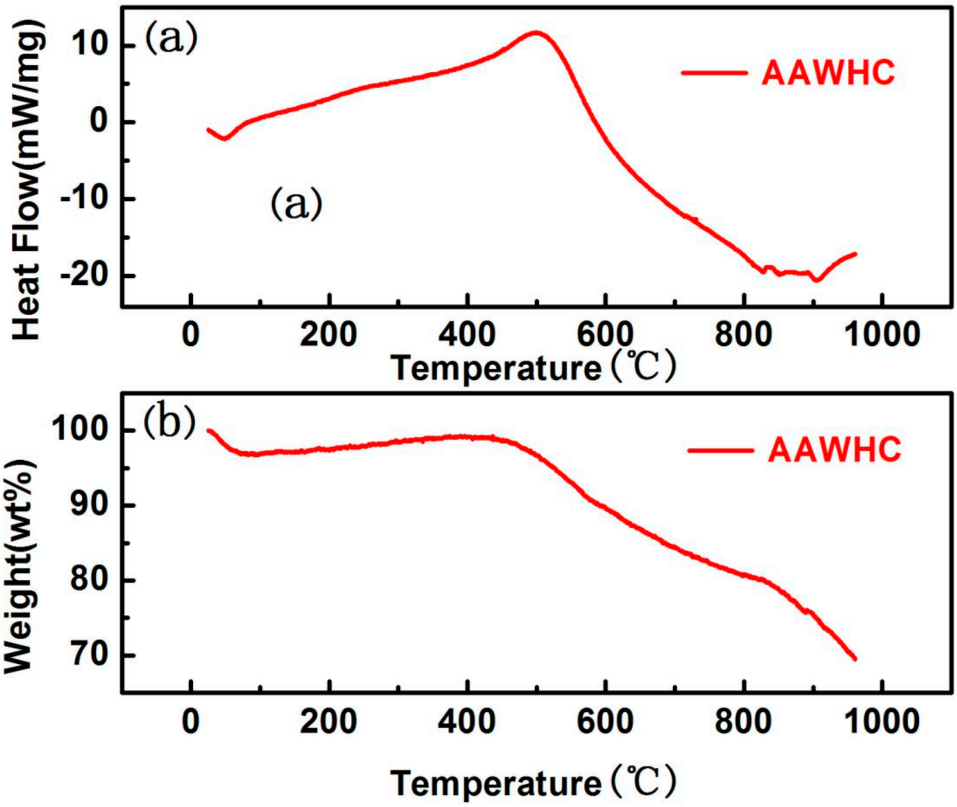

Figure S2. (**a**) DSC curve of AAWHC. (**b**) TG curve of AAWHC.

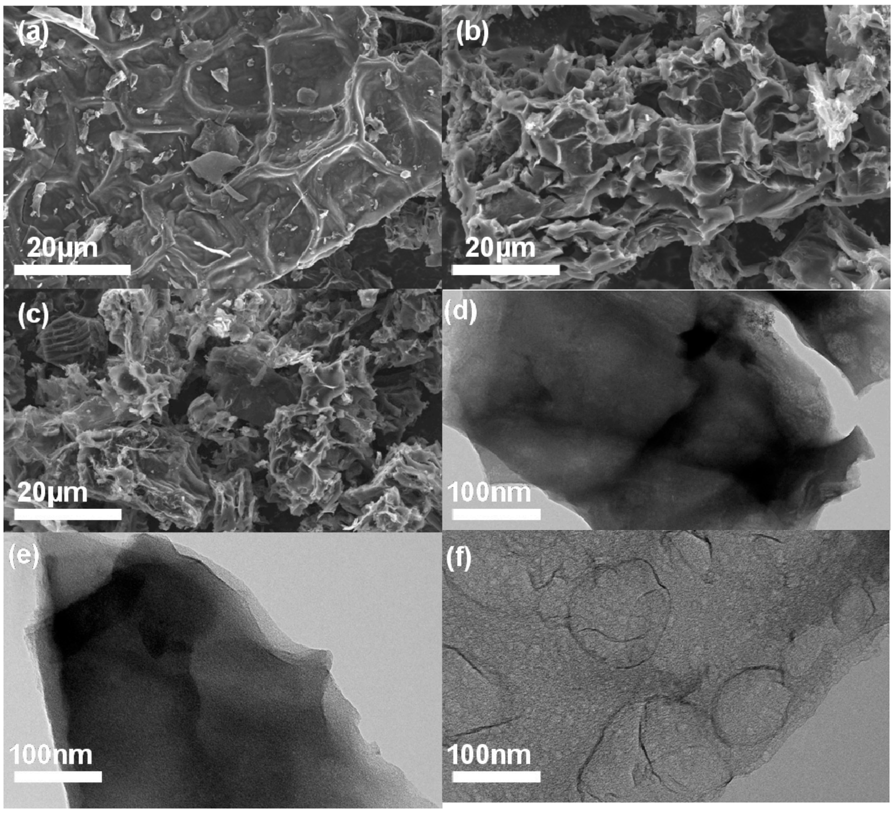

Figure S3. (**a**) SEM image of WHC. (**b**) SEM image of AWHC.

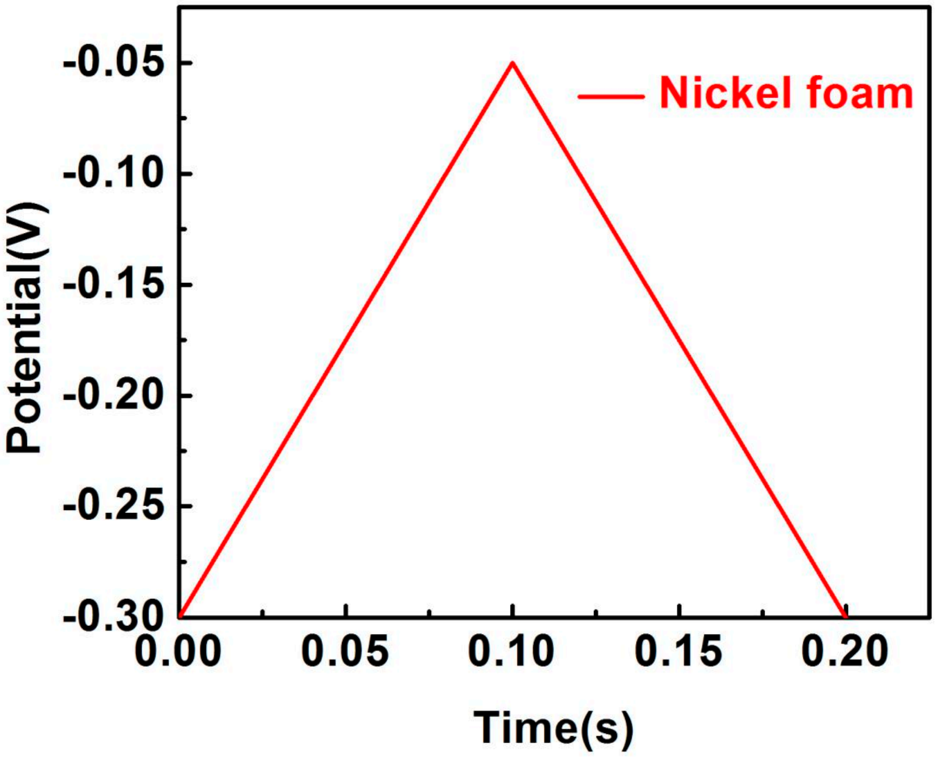

Figure S4. GCD curve of the pure nickel foam at 1 A g^{-1}.

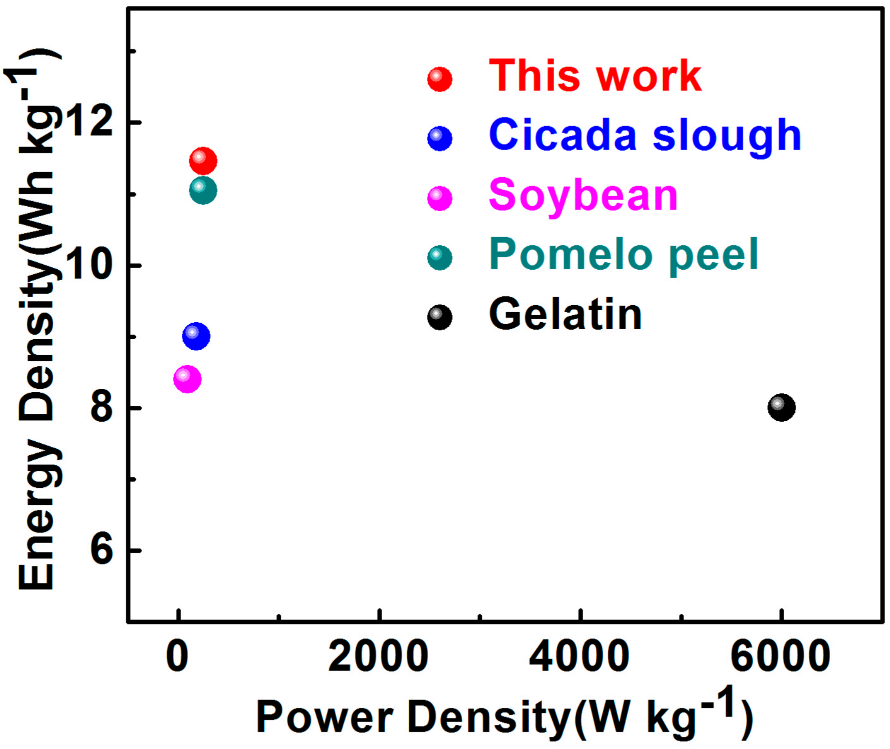

Figure S5. Energy density comparison with some reported biomass carbon-derived electrodes.

Table S1. Comparison with the latest reports of biomass carbon.

Raw Materials	Current Density (A g–1)	Specific Capacitance (F g–1)	Ref.
bamboo char	0.5	222	[1]
Cashmere	1	236	[2]
Pomelo peel	0.5	240	[3]
Silk Proteins	0.5	264	[4]
cattail wool	1	314	[5]
Camellia oleifera shell	0.5	315	[6]
Corn straw	0.3	327	[7]
soybean	0.5	330	[8]
Auricularia	1	339	[9]
Cicada slough	1	355	[10]
**water hyacinth**	**1**	**374**	**This work**

Reference

1. Gong Y; Li D; Luo C; Fu Q; Pan C. Highly porous graphitic biomass carbon as advanced electrode materials for supercapacitors. *Green Chemistry*, 2017, 19, 4132-4140; Doi:10.1039/c7gc01681f.

2. Zhou L; Cao H; Zhu S; Hou L; Yuan C. Hierarchical micro-/mesoporous N- and O-enriched carbon derived from disposable cashmere: a competitive cost-effective material for high-performance electrochemical capacitors. *Green Chemistry*, 2015, 17, 2373-2382; Doi:10.1039/c4gc02032d.

3. Wang Z; Tan Y; Yang Y; et al. Pomelo peels-derived porous activated carbon microsheets dual-doped with nitrogen and phosphorus for high performance electrochemical capacitors. *Journal of Power Sources*, 2018, 378, 499-510; Doi:10.1016/j.jpowsour.2017.12.076.

4. Yun Y.S.; Cho S.Y.; Shim J; et al. Microporous carbon nanoplates from regenerated silk proteins for supercapacitors. Adv Mater, 2013, 25, 1993-1998; Doi:10.1002/adma.201204692.

5. Su X.L.; Jiang S; Zheng G.P.; Zheng X.C.; Yang J.H.; Liu Z.Y High-performance supercapacitors based on porous activated carbons from cattail wool. *Journal of Materials Science*, 2018, 53,9191-9205; Doi:10.1007/s10853-018-2208-5.

6. Liang J; Qu T; Kun X; et al. Microwave assisted synthesis of camellia oleifera shell-derived porous carbon with rich oxygen functionalities and superior supercapacitor performance. *Applied Surface Science*, 2018, 436; 934-940. Doi:10.1016/j.apsusc.2017.12.142.

7. Qiu Z; Wang Y; Bi X; et al. Biochar-based carbons with hierarchical micro-meso-macro porosity for high rate and long cycle life supercapacitors. *Journal of Power Sources*, 2018, 376, 82-90; Doi:10.1016/j.jpowsour.2017.11.077.

8. Zhao H; Xing B; Zhang C; et al. Efficient synthesis of nitrogen and oxygen co-doped hierarchical porous carbons derived from soybean meal for high-performance supercapacitors. *Journal of Alloys and Compounds*, 2018, 766, 705-715; Doi:10.1016/j.jallcom.2018.06.267.

9. Long C; Chen X; Jiang L; Zhi L; Fan Z. Porous layer-stacking carbon derived from in-built template in biomass

for high volumetric performance supercapacitors. *Nano Energy*, 2015, 12, 141-151; Doi:10.1016/j.nanoen.2014.12.014.

10. Jia H; Sun J; Xie X; Yin K; Sun L. Cicada slough-derived heteroatom incorporated porous carbon for supercapacitor: Ultra-high gravimetric capacitance. *Carbon*, 2019, 143, 309-317; Doi:10.1016/j.carbon.2018.11.011.
